# From conservation genetics to conservation genomics: a genome-wide assessment of blue whales (*Balaenoptera musculus*) in Australian feeding aggregations

**DOI:** 10.1098/rsos.170925

**Published:** 2018-01-31

**Authors:** Catherine R. M. Attard, Luciano B. Beheregaray, Jonathan Sandoval-Castillo, K. Curt S. Jenner, Peter C. Gill, Micheline-Nicole M. Jenner, Margaret G. Morrice, Luciana M. Möller

**Affiliations:** 1 Molecular Ecology Lab, College of Science and Engineering, Flinders University, GPO Box 2100, Adelaide, South Australia 5001, Australia; 2 Cetacean Ecology, Behaviour and Evolution Lab, College of Science and Engineering, Flinders University, GPO Box 2100, Adelaide, South Australia 5001, Australia; 3 Centre for Whale Research, PO Box 1622, Fremantle, Western Australia 6959, Australia; 4 Blue Whale Study, C/- Post Office, Narrawong, Victoria 3285, Australia; 5 School of Life and Environmental Sciences, Deakin University, PO Box 423, Warrnambool, Victoria 3280, Australia

**Keywords:** cetaceans, double-digest restriction-site associated DNA sequencing, ecological genomics, molecular ecology, non-model organism, population genomics

## Abstract

Genetic datasets of tens of markers have been superseded through next-generation sequencing technology with genome-wide datasets of thousands of markers. Genomic datasets improve our power to detect low population structure and identify adaptive divergence. The increased population-level knowledge can inform the conservation management of endangered species, such as the blue whale (*Balaenoptera musculus*). In Australia, there are two known feeding aggregations of the pygmy blue whale (*B. m. brevicauda*) which have shown no evidence of genetic structure based on a small dataset of 10 microsatellites and mtDNA. Here, we develop and implement a high-resolution dataset of 8294 genome-wide filtered single nucleotide polymorphisms, the first of its kind for blue whales. We use these data to assess whether the Australian feeding aggregations constitute one population and to test for the first time whether there is adaptive divergence between the feeding aggregations. We found no evidence of neutral population structure and negligible evidence of adaptive divergence. We propose that individuals likely travel widely between feeding areas and to breeding areas, which would require them to be adapted to a wide range of environmental conditions. This has important implications for their conservation as this blue whale population is likely vulnerable to a range of anthropogenic threats both off Australia and elsewhere.

## 1. Introduction

Next-generation sequencing has brought about an orders-of-magnitude increase in the number of DNA markers feasible for studies of non-model organisms. Large, genome-wide datasets boost the power of traditional analyses that use small numbers of neutral loci to evaluate population structure, and allow the investigation of the small proportion of loci in the genome that exhibit ecologically relevant adaptation [1–3]. We can now assess with confidence whether putative populations that have shown no population structure based on traditional ‘previous-generation sequencing’ genetic datasets might actually have low, but biologically relevant, population structure. Low levels of population structure could be a result of recent divergence, high gene flow, or low genetic drift due to large effective population size. Genotyping-by-sequencing datasets have been used recently to resolve such low population structure [4–6]. These datasets may also be used to detect adaptive divergence even when there is negligible neutral differentiation [7,8]. Such adaptive divergence should be taken into account when making management decisions, such as when delineating units for conservation [9].

Marine mammals have the ecological attributes that can give rise to both low levels of neutral structure and adaptive divergence. They are active, long-distance dispersers with few barriers to movement, but often have neutral population structure that is associated with environmental requirements or preferences [10]. For baleen whales, population structure is intrinsically related to their temporally changing environmental requirements: they typically breed at lower latitudes in the winter and migrate to feed at higher latitudes in the summer [11]. This means feeding grounds can comprise one or more populations that seasonally segregate to breed. There may be site fidelity to the calving ground where they were born, or to a particular feeding ground through cultural learning of its location from their mother in the first year of life [12,13]. Divergent environmental conditions between localities, such as feeding or breeding grounds, also give rise to the possibility of adaptive divergence. Knowledge of neutral and adaptive population structure is expected to aid the conservation of threatened species [9], and may particularly aid the conservation of baleen whales given many populations are still recovering from twentieth century whaling, and it has been difficult to appropriately define their management units [14]. Marine mammal genomic studies to date have focused on phylogenomics and comparative genomics, and are only now beginning to investigate questions in population genomics [15].

Here, we conduct a population genomic study on blue whales (*Balaenoptera musculus*) that feed off Australia. These are of the pygmy blue whale subspecies (*B. m. brevicauda*) and were founded relatively recently, likely around the time of the Last Glacial Maximum [16,17]. There are two known primary feeding grounds in Australia: the Perth Canyon and adjacent waters off Western Australia [18], and the Bonney Upwelling and adjacent waters off South Australia and Victoria in southern Australia [19]. Our previous genetic study found no evidence of population structure between or within these feeding aggregations, although we only used a traditional genetic dataset of 10 microsatellites and the mtDNA control region [20]. This also means that any maternally mediated site fidelity to aggregation areas is too recent to be detectable in mtDNA—as may be expected given the recent founding of the population [16]—or is of insufficient strength to give a mtDNA signal. The blue whales occupying the feeding aggregation areas likely migrate along Western Australia and, for those travelling from the Bonney Upwelling, along or offshore of southern Australia to overwinter, and presumably breed, in and around Indonesia [21–25].

If whales show fidelity to a particular feeding ground, environmental differences between the feeding grounds may favour adaptive differentiation. Upwelling in the austral summer results in high primary productivity in both feeding grounds, driven by different weather and ocean-current systems [18,26]. This results in complex environmental differences between the feeding grounds that are related to, for example, depth, temperature, salinity and primary productivity [18,21,27,28]. Each feeding ground also has a different species of krill—the focal prey item of blue whales—which typically occur at different depths of the water column during the day. *Euphausia recurva* occurs at the Perth Canyon at depths of around 300–600 m and the whales dive to those depths to forage [18], whereas *Nyctiphanes australis* occurs in the Bonney Upwelling in shallower waters of the continental shelf, frequently forming surface swarms, and the blue whales there are sighted in waters with a mean depth of only 100 m [19,21,29,30]. This may lead to adaptively different feeding strategies between blue whales occupying different feeding grounds, including divergent diving behaviour and physiology. Any adaptive divergence would be expected to have evolved relatively quickly from standing genetic variation given that this population was recently founded, likely from the genetically diverse Antarctic blue whale (*B. m. intermedia*) [16].

We use a genomic dataset of 8294 filtered single nucleotide polymorphisms (SNPs) derived from double-digest restriction-site associated DNA sequencing (ddRAD) to carry out a genome-wide assessment of neutral and adaptive population structure for the pygmy blue whales feeding off Australia. The neutral population structure could form one of three possible patterns [11,20]: (1) each feeding ground is occupied by a different population (i.e. neutral divergence of the feeding aggregations); (2) there is sharing of feeding grounds by multiple populations that segregate when breeding (i.e. neutral structure within one or both feeding aggregations); (3) both feeding grounds are shared by the same population (i.e. no neutral structure within or between feeding aggregations). Attard *et al.* [20] used microsatellites to infer the third scenario, but there may be low population structure (i.e. scenario 1 or 2) that could be detected using the greater power of a genomic dataset. We also assessed whether there was adaptive divergence between feeding aggregations, which may occur regardless of whether there is neutral divergence of the feeding aggregations (i.e. scenario 1 versus scenario 3) because strong selection can overcome gene flow [7,31]. It may occur even if populations share feeding grounds (i.e. scenario 2) because neutral lineages do not necessarily reflect adaptive structure [32]. Adaptive divergence associated with feeding ground origin may occur due to known differences in environmental characteristics between feeding grounds combined with cultural learning of feeding ground locations and long-term site fidelity to feeding grounds, with both known to occur in other baleen whales [12,13]. This is the first time that spatial variation in adaptive diversity of blue whales has been examined. We discuss the biological and conservation relevance of our findings in the context of blue whale research in other fields.

## 2. Material and methods

### 2.1. Data collection

**Figure 1.**
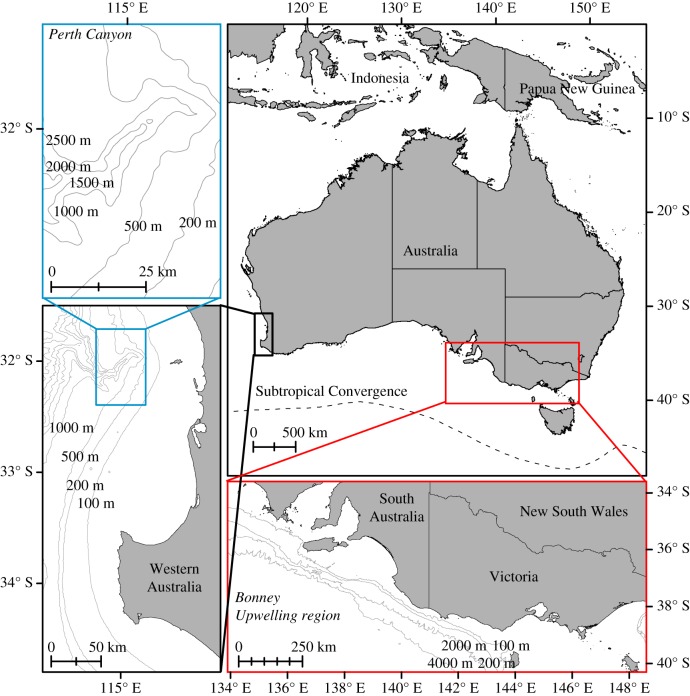
Location of the two known feeding aggregations of pygmy blue whales off Australia: the Perth Canyon (blue) and the Bonney Upwelling (red).

Biopsy samples of blue whales were collected from the Perth Canyon (*n* = 72) and Bonney Upwelling (*n* = 38) feeding aggregations in Australia (figure 1) from 1995 to 2012 in October to April, predominately using a Paxarms (New Zealand) remote biopsy rifle [33]. This excludes our previously detected resamples from microsatellites [17,20], none of which were between feeding grounds, and one of our previous studies also confirmed that all sampled blue whales were of the pygmy blue whale subspecies [17]. Samples were preserved in either 20% dimethylsulfoxide saturated with NaCl or 70–100% ethanol. DNA was extracted using a modified salting-out protocol [34]. The genomic DNA was checked for quality using a spectrophotometer (NanoDrop, Thermo Scientific), integrity using 2% agarose gels and quantity using a fluorometer (Qubit, Life Technologies). From those we chose a subset of samples for library preparation: 42 samples from the Perth Canyon and 36 from the Bonney Upwelling. Libraries were prepared in-house following the ddRAD protocol of Peterson *et al.* [35] and modified as described in Brauer *et al.* [36]. Sequencing was performed paired-end 100 bp using 36 samples per lane in three lanes of a HiSeq 2000 Illumina (i.e. 30 samples across the lanes were not part of the current study). Resulting reads were processed using the *de novo* pipeline of STACKS 1.29 [37,38] to produce a final SNP dataset (electronic supplementary material). The final SNP dataset consisted of SNPs that were in at least 70% of samples and had a minor allele frequency of at least 0.05. Only the first SNP from each polymorphic ddRAD locus was used to minimize linkage disequilibrium.

We note that the traditional genetic dataset of 10 microsatellites from Attard *et al.* [20] to assess genetic structure in Australian feeding aggregations of blue whales has since expanded into 20 microsatellites used by Attard *et al.* [16,17], with neither of the latest studies re-assessing genetic structure in Australia. We therefore conducted such analyses on this expanded but still relatively small 20 marker dataset to determine whether it could detect population structure, but it still showed little evidence for genetic structure (electronic supplementary material).

### 2.2. Genetic variation and population structure

Genetic variation for each feeding aggregation was determined by calculating the percentage of polymorphic loci, mean observed heterozygosity and mean unbiased expected heterozygosity using GENALEX 6.502 [39,40]. Population structure between feeding aggregations was assessed as pairwise genetic differentiation (*F*_ST_) using ARLEQUIN 3.5.1.2 [41] (significance assessed by 10 000 permutations), principal component analysis (PCA) implemented in ADEGENET 2.0.0 [42] (missing data imputed using ‘mean’) in R 3.2.3 [43], discriminant analysis of principal components (DAPC) [44] also implemented in ADEGENET (the optimal number of clusters for DAPC was determined using Bayesian information criterion (BIC)) and a Bayesian clustering analysis carried out in fastSTRUCTURE 1.0 [45]. FastSTRUCTURE was implemented with the default settings (simple prior, 10^−6^ convergence criterion) and testing values of *K* from one to four. The values of K*_ε_ (model complexity that maximizes marginal likelihood) and K*_øc_ (model components used to explain structure in data) were used to infer the number of clusters.

To confirm the greater power of the genomic dataset, statistical power was estimated using a large SNP dataset version of POWSIM [46] following the same parameters as Attard *et al.* [20]; this version is obtainable from Nils Ryman, and allows up to 32 000 loci, two alleles per locus and 40 populations, whereas the standard version (currently 4.1) allows up to 50 loci, 50 alleles per locus and 30 populations. While the program can use both *χ*^2^ and Fisher's exact test as statistics, only the former is appropriate for large SNP datasets due to the latter behaving poorly when combining *p*-values across many loci and when the contingency tables are small (here the contingency tables are two populations × two alleles) [47,48], and so only *χ*^2^ was used.

### 2.3. Adaptive divergence

Two approaches were used to detect any signal of adaptive divergence. First, we tested for markers potentially under directional selection using *F*_ST_ outlier methods: the Bayesian method of BAYESCAN 2.1 [49] and the method of FDIST2 [50] as implemented in LOSITAN [51] (default number of simulations (50 000) with addition of ‘neutral’ and ‘force’ mean *F*_ST_ options). We used a false discovery rate (FDR) of 0.1 to correct for type I errors from multiple testing. Both these programs detect simultaneously loci under either balancing or directional selection. However, as loci under balancing selection are expected to have lower differentiation than neutral loci (and loci under directional selection have greater differentiation), it is problematic to discern balancing selection in biological systems with low or no neutral structure [52]. Therefore, from these programs we only extracted loci that showed evidence for directional selection (and considered those showing evidence for balancing selection to be neutral loci).

Second, we implemented a machine-learning method, Random Forest [53], to assess whether there is adaptive divergence between the feeding aggregations and, if so, identify markers associated with this divergence. Random Forest attempts to classify individuals into groups based on putative predictor variables, in this case into feeding aggregations based on potentially informative SNP loci. The subset of loci found to be informative for this allocation are considered putative adaptive loci, especially given no evidence of neutral structure in this dataset (see §3.2). Random Forest has been successfully used for detecting adaptive divergence in previous studies, especially in situations of no neutral differentiation [7,54]. It is also particularly suited to datasets with a large number of variables, or loci, but a small number of samples [55], and for detecting adaptive signal associated with polygenic traits [56]. The Random Forest was created with 10 000 trees, the proximity and importance options and the number of randomly chosen variables (i.e. loci) considered in each split of the tree (mtry) as the default, which is the square root of the number of variables (i.e. square root of the number of loci). For the latter, we also trialled a higher value of 1000 because higher values provide a greater chance of a tree including informative variables (i.e. loci under selection) when the dataset has relatively few such variables. Missing data were imputed—which is a requirement of the analysis—in GENODIVE 2.0b27 [57] by conservatively (i.e. expected to find fewer loci putatively under selection) drawing alleles from the allele frequency distribution of the entire dataset rather than for each feeding aggregation separately.

A randomization procedure was used to assess the possibility of false positives (i.e. type I errors) in analyses that found evidence of putatively adaptive loci (i.e. FDIST2; see §3.3). This involved randomizing samples between the feeding aggregations 10 times, and running each of the 10 randomizations in analyses that detected putatively adaptive loci using the same parameters as the non-randomized dataset. The amount of loci showing signals of ‘adaptive divergence’ in the randomized datasets is an indication of the amount of false positives produced by the analysis in this study system. Samples were randomized using RandomizeLines.py for Notepad++ Python Scripting plugin (https://github.com/ethanpil/npp-randomizelines).

## 3. Results

### 3.1. Data collection

A total of 510 337 967 forward reads were generated in three lanes of the Illumina platform. After demultiplexing and quality filtering, a range from 336 923 to 3 498 349 reads with an average of 1 698 149 reads were obtained per individual sequenced for the current study, totalling 132 455 605 reads. *De novo* assembly and catalogue filtering to generate a SNP dataset revealed that seven samples from the Bonney Upwelling and three from the Perth Canyon had a relatively high (greater than 40%) amount of missing data. They were therefore removed and the catalogue re-filtered. This resulted in 25 766 ddRAD loci containing 11 194 SNPs in 68 samples from the Bonney Upwelling (*n* = 29) and the Perth Canyon (*n* = 39). A final dataset of 8294 SNPs was obtained by extracting only the first SNP from each polymorphic ddRAD locus to remove SNPs that are likely in linkage disequilibrium.

### 3.2. Population structure

**Figure 2.**
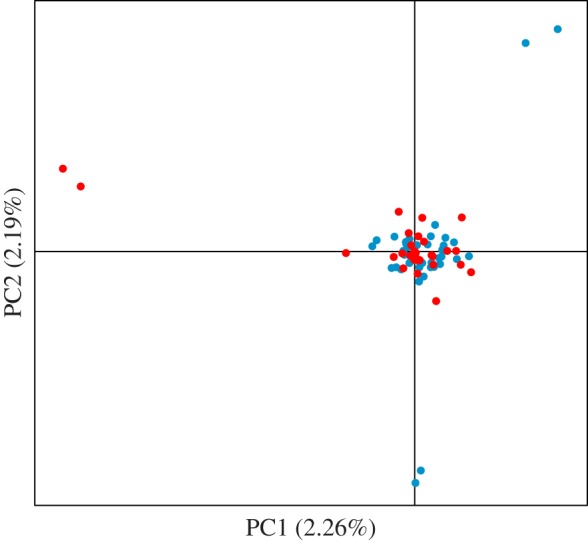
PCA from 8294 SNPs of pygmy blue whales from the Australian feeding aggregations (red, Bonney Upwelling; blue, Perth Canyon).

**Figure 3.**
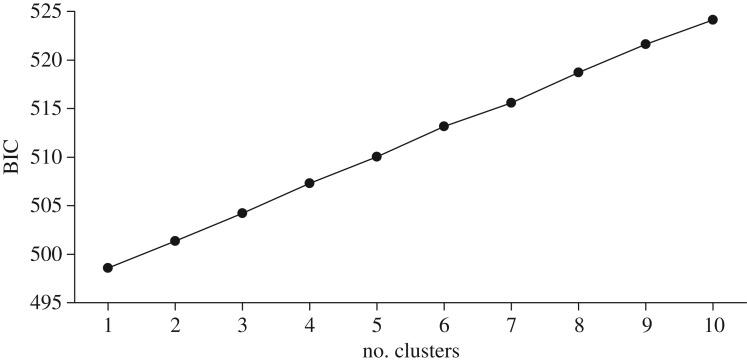
Inference of the number of genetic clusters using Bayesian information criterion (BIC) in the R package ADEGENET for DAPC analysis. The optimal clustering solution corresponds to the lowest BIC, which in this case is one cluster.

The Bonney Upwelling and Perth Canyon samples had similar levels of genetic variation: 99.3% and 99.8% polymorphic loci, 0.205 (standard deviation (s.d.) 0.134) and 0.195 (s.d. 0.122) mean observed heterozygosity, and 0.327 (s.d. 0.144) and 0.326 (s.d. 0.139) mean expected heterozygosity, respectively. There was no evidence of genetic differentiation based on *F*_ST_ and the associated permutation test (*F*_ST_ = 0.004, *p* = 0.237). The PCA showed complete overlap of whales from the two feeding aggregations (figure 2); the three pairs of individuals outside the cluster are pairs of highly related individuals [58]. The optimal clustering solution determined in ADEGENET for DAPC analysis was one cluster based on BIC (figure 3). The Bayesian clustering analysis inferred one genetic cluster, with both fastSTRUCTURE statistics selecting one as the most likely *K*. The power analysis confirmed that the 8294 SNP dataset was more powerful than the 10 microsatellite dataset of Attard *et al.* [20] (or a 20 microsatellite dataset; see the electronic supplementary material): Attard *et al.* [20] found that 10 microsatellites could detect a *F*_ST_ of greater than or equal to 0.0151 (*t* = 60) with greater than or equal to 95% confidence (96.2% *χ*^2^; 95.5% Fisher's exact test); here we found that 8294 SNPs could detect a *F*_ST_ of greater than or equal to 0.0010 (*t* = 4) with greater than or equal to 95% confidence (100% *χ*^2^; Fisher's exact test is inappropriate for large SNP datasets, see §2.2).

### 3.3. Adaptive divergence

**Figure 4.**
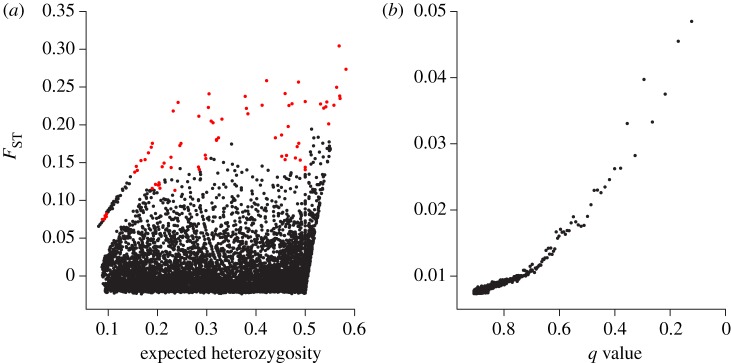
*F*_ST_ outlier test results of (*a*) FDIST2 and (*b*) BAYESCAN, showing loci detected as putatively under directional selection based on a FDR of 0.1 (red circles; only FDIST2 found loci putatively under selection).

The *F*_ST_ outlier methods of FDIST2 and BAYESCAN identified 82 and no SNPs putatively under selection, respectively (figure 4). The machine-learning method of Random Forest detected no SNPs putatively under selection. Specifically, it seems that individuals were biased towards being classified in Random Forest into the group with the greater number of samples, the Perth Canyon. Random Forest is expected to classify individuals into the class of greater sample size in datasets that are uninformative for distinguishing between classes, here the two different feeding aggregations, in order to minimize the overall error rate [59]. Trial runs using a subset of individuals from the Perth Canyon to equalize sample numbers did not improve performance, showing an error rate of at least 50%. As the only method to show evidence of putatively adaptive loci was FDIST2, a randomization procedure was conducted for FDIST2 to assess whether the detected outliers are likely false positives. The average number of outlier loci detected by FDIST2 in the 10 randomized datasets was 70 (s.d. = 24, range = 36–102). This means it is likely that most if not all of the 82 outlier loci detected by FDIST2 in the non-randomized dataset are false positives.

## 4. Discussion

We successfully produced the first population genomic dataset for blue whales: 8294 filtered SNPs for the two feeding aggregations of pygmy blue whales in Australia. A series of analyses found no evidence of neutral structure, strongly suggesting that whales from these aggregations are a single genetic population. The lack of neutral structure in this large SNP dataset indicates that this is a biologically relevant finding rather than due to the use of few loci and associated low power, or associated poor representation of the genome, which could have been the case with the previous 10 microsatellite dataset [20] or 20 microsatellite dataset (see electronic supplementary material). This is supported by a power analysis of each dataset. In terms of adaptive structure, the signal in this dataset is limited, with only one (FDIST2) of the three methods (FDIST2, BAYESCAN, Random Forest) detecting putatively adaptive loci. It is highly likely that many if not all of the detected putatively adaptive loci from FDIST2 are false positives because the number of outliers detected (82) was within 1 s.d. of the number of outliers detected when samples were randomized between the feeding aggregations (mean = 70, s.d. = 24). Differences in the methods to detect adaptive divergence as well as the particular demographic characteristics of the study system can interact to generate different results and type I (and II) error rates between methods [60,61]. While both FDIST2 and BAYESCAN are *F*_ST_ outlier methods, the former uses a coalescent-based approach [50] and the latter a Bayesian approach [49] with the population model differing between these methods; Random Forest instead is a machine-learning approach with no population model [53]. If there is adaptive divergence between feeding aggregations, it is likely weak or at loci not sampled here. Altogether, there is no evidence of neutral structure and negligible evidence of adaptive structure in Australian feeding aggregations of pygmy blue whales.

We propose that the lack of neutral divergence and limited or no adaptive divergence between feeding aggregations is because individuals travel widely between feeding areas in Australia (and potentially feeding areas elsewhere) during the austral summer and to the Indonesian region during the austral winter. This would require them to be adapted to a wide range of environments as well as feeding strategies associated with the varied behaviour of krill (see Introduction for background information related to Australia). Movements between localities may also be promoted by the inter-annual variability in the density and distribution of blue whale prey [62]. Our proposal is supported not only by the neutral and adaptive findings here, but evidence from other fields. This wider evidence includes, first, the near-continuous distribution of blue whales based on sightings, strandings and catch records from the Bonney Upwelling, to the Perth Canyon, along the coast of Western Australia to Indonesia, and across the southern Indian Ocean [22]. Second, satellite tagging of blue whales at the Perth Canyon has shown movement to surrounding areas in southwestern Australia and migration along Western Australia to Indonesia, where they occupy relatively productive upwelled waters during the winter breeding season [24]. Third, the unique call of this population has been recorded in and around the austral summer off southwestern Australia [23,25,63,64], at the Bonney Upwelling [23,65], in the southern Indian Ocean off Amsterdam Island [66] and at the sub-Antarctic Crozet Islands [67]. It has also been recorded during the expected migratory period off Western Australia [22,68] and in the austral winter in Indonesia [25]. Fourth, aerial surveys in the Bonney Upwelling region indicate movements of blue whales from the west to the Bonney Upwelling for summer feeding [21]. Fifth, dive records from tags in Australia suggest that blue whales can switch between shallow and deep dives during summer feeding (Möller *et al.* unpublished data), and in Indonesia suggest feeding behaviour even during the winter breeding season (Kahn unpublished data) [24], with other populations also showing context-dependent diving behaviour [69]. Their relatively thin blubber layer [70] also suggests they do not fast like other baleen whales. Altogether, the data from the genome, environmental observations, sightings, satellite tagging, acoustics and dive records suggest that this population of blue whales may visit diverse, widespread areas for feeding during the austral summer, including perhaps the southern Indian Ocean and sub-Antarctic region, and travel to winter breeding grounds in the Indonesian region where they may also feed. This could be confirmed through photo-identification to determine whether there is site fidelity of individuals to particular feeding areas, and through additional satellite tagging efforts to clarify movement patterns and the geographical range of the population.

This population is not believed to reach regions as distant as the western Indian Ocean, western Pacific Ocean and south of the Antarctic Polar Front. A different, unique call type of blue whales predominates at each of these locations [23,25,64,66,67,71], likely representing different lineages. However, confirmation of the degree of connectivity of the study population with these three areas requires genetic or, ideally, genomic studies. The only focused comparisons have been to the Antarctic using traditional genetic datasets, where the Antarctic blue whale subspecies (*B. m. intermedia*) predominates during the feeding season [17,72]. Although, there was also evidence for a recent increase in the connectivity of pygmy blue whales (i.e. the subspecies of the study population in this paper) to Antarctic blue whales likely due to human impacts [17]. Antarctic blue whales are thought to migrate to lower latitudes for the winter breeding season [17,22,73,74] but have also been recorded year-round in the Antarctic [22,73–76]. While adaptive differences were negligible at the fine scale of the current study, there is the potential for adaptive differences between the Antarctic and pygmy subspecies, as well as other recognized blue whale subspecies, especially given evidence of morphological differences between recognized subspecies and even between putative populations of the blue whale subspecies in the Northern Hemisphere (*B. m. musculus*) [77–80].

Our study is the first assessment of population genomic structure or adaptive variation in baleen whales. It adds to the growing studies using thousands of SNPs for assessing potential low neutral structure or adaptive divergence in species with high dispersal capabilities [4,5,7,8]. Specifically, we showed that the blue whales in the two Australian feeding aggregations belong to one population and we found negligible evidence of adaptive divergence between the aggregations. We propose that these findings are due to the blue whales travelling between feeding areas, as well as travelling thousands of kilometres when migrating to breed and perhaps also to feed in the Indonesian region, requiring them to be flexible with regard to different environmental conditions and feeding strategies. This has important implications for their conservation: it means that one population, defined both in terms of neutral and adaptive variation, likely travels through waters off Australia and elsewhere; individuals from this population are likely impacted upon by different anthropogenic threats throughout their range; and impacts of geographically restricted threats may have a flow-on effect to the entire population. We suggest that future work should use genotyping-by-sequencing datasets for refining population structure knowledge of this endangered species worldwide and for assessing adaptive divergence between morphologically divergent subspecies and other putative populations of blue whales.

## Supplementary Material

Electronic supplementary material

## Data accessibility

SNP genotypes are available under Attard *et al.* [58] in Dryad entry http://dx.doi.org/10.5061/dryad.t8ph5 (in the SNP data file, the first 29 individuals are from the Bonney Upwelling and the last 39 individuals are from the Perth Canyon). The microsatellite genotypes for the comparative 20 microsatellite dataset are under Attard *et al.* [17] in Dryad entry http://dx.doi.org/10.5061/dryad.8m0t6, with the exception of one additional sample that is under the Dryad entry of Attard *et al.* [58].
