# Supplementary material for: From conservation genetics to conservation genomics: a genome-wide assessment of blue whales (Balaenoptera musculus) in Australian feeding aggregations
Source: R Soc Open Sci. 2018 Jan 31;5(1):170925. doi: 10.1098/rsos.170925 (PMC5792883; doi:10.1098/rsos.170925)
Supplement: Electronic supplementary material [file rsos170925supp1.docx]

**SUPPLEMENTARY MATERIAL**

for the article

**From conservation genetics to conservation genomics: a genome-wide assessment of blue whales (*Balaenoptera musculus*) in Australian feeding aggregations**

**DATA COLLECTION**

The reads from HiSeq 2000 Illumina were demultiplexed and filtered for quality using the “process_radtags” pipeline of STACKS 1.29 (Catchen *et al.* 2013; Catchen *et al.* 2011). Barcodes and RAD-tags on the read were allowed up to two mismatches each before a read was discarded. Reads were trimmed to 68 bp (excluding barcode and RAD-tag) based on trial runs that showed an increase in the number of SNPs called after this value, which is likely due to decreasing read quality and associated misgenotyping. Phred scores were then permitted down to an average of 20 within a sliding window of 15% before a read was discarded.

In the absence of a reference genome for this species, ddRAD loci were assembled *de novo* using the “denovo_map” pipeline of STACKS. Only forward reads were used for assembly. Different parameter combinations were tested for the number of nucleotides allowed to be different between stacks (identical reads) at a locus within an individual (-M 3-4; -N was kept as default of M+2) and across individuals (-n 2-5), which resulted in little difference in the final number of SNPs (maximum 184 difference). Therefore, we used a value of three for both parameters. The minimum number of reads required to form a stack was the default of three (-m), the maximum number of stacks permitted at a locus was the default of three (--max_locus_stacks), the upper bound for epsilon was 0.03 (--bound_high), and the chi-square significance level to call a heterozygote or homozygote was 0.1 (--alpha). The deleveraging algorithm was implemented to resolve over-merged ddRAD loci (-d) and the removal algorithm to drop highly-repetitive stacks (-r).

The “populations” pipeline of STACKS was used to filter ddRAD loci and SNPs from the assembled *de novo* catalog to produce a final SNP dataset for subsequent analyses. DdRAD loci needed to be in at least 70% of samples (-r), SNPs needed a minor allele frequency of at least 0.05 (-a), and individuals needed a depth of coverage of at least six at a locus (-m). Custom scripts were used to check the distribution of SNPs across ddRAD loci from the outputted vcf file (as an increase in SNPs towards the end of ddRAD loci indicates potential misgenotyping) and to extract the first SNP from each ddRAD locus to minimize linkage disequilibrium. Individuals found to have more than 40% missing data were removed from the dataset by re-running the “populations” pipeline and then the custom scripts without these individuals. PGDSpider 2.1.0.0 (Lischer & Excoffier 2012) and CREATE 1.37 (Coombs *et al.* 2008) were used to convert the dataset into formats required for other programs.

**POPULATION STRUCTURE: TRADITIONAL MICROSATELLITES**

We assessed genetic structure in Australian feeding aggregations of blue whales using the traditional genetic dataset of 20 microsatellites from Attard *et al.* (2012; 2015) with the addition here of one new sample (i.e. Bonney Upwelling = 38; Perth Canyon = 72). This was to confirm that the increase of 10 microsatellites from the study by Attard *et al.* (2010) still found a lack of genetic structure. The microsatellite dataset passed checks during these previous studies for null alleles, genotyping errors, short allele dominance, and linkage disequilibrium. Nine of the 20 microsatellites are those used by Attard *et al.* (2010) to assess population structure in the Australian feeding aggregations.

Genetic variation for each feeding aggregation was determined by calculating the number of alleles, mean observed heterozygosity and mean unbiased expected heterozygosity using GENALEX 6.502 (Peakall & Smouse 2006, 2012). Pairwise genetic differentiation (*F*_ST_) was assessed using ARLEQUIN 3.5.1.2 (Excoffier & Lischer 2010) (significance assessed by 10 000 permutations) and Bayesian clustering analysis was conducted using STRUCTURE 2.3.4 (Pritchard *et al.* 2000). The STRUCTURE analysis was implemented using the admixture model of ancestry, the correlated allele frequency model (Falush *et al.* 2003), and without sampling locations as priors (Hubisz *et al.* 2009) (burn-in of 100 000 iterations then runs of 10^6^). Ten independent runs were conducted for each value of *K* from one to four, where *K* is the number of inferred genetic clusters. The most meaningful value of *K* was determined from the probability of the data for each tested value of *K*, as extracted from the runs using STRUCTURE HARVESTER 0.6.94 (Earl & vonHoldt 2012). The Δ*K* method (Evanno *et al.* 2005) was not used as it can only detect a true *K* that is greater than one. Statistical power was estimated using POWSIM 4.1 (Ryman & Palm 2006) following the same parameters as Attard *et al.* (2010).

The Bonney Upwelling and Perth Canyon had similar levels of genetic variation: respectively, 5.3 and 5.9 mean number of alleles, 0.576 (standard deviation (s.d.) 0.197) and 0.615 (s.d. 0.199) mean observed heterozygosity, and 0.585 (s.d. 0.199) and 0.604 (s.d. 0.185) mean expected heterozygosity. There was very low but significant genetic differentiation based on *F*_ST_ and the associated permutation test for microsatellites (*F*_ST_ = 0.005, *P* = 0.014). Given no concordance of this result with any other analysis, we do not think it is biologically relevant. The PCA showed complete overlap of whales from the two feeding aggregations (figure S1). The Bayesian clustering analysis inferred one genetic cluster, with the likelihood in STRUCTURE across different values of *K* showed a peak at a *K* of one (-5323.04 (s.d. 0.49)). The power analysis found that the 20 microsatellites could detect a *F*_ST_ of greater than or equal to 0.0067 (*t* = 27) with greater than or equal to 95% confidence (96.5% chi-square; 95.4% Fisher’s exact test).


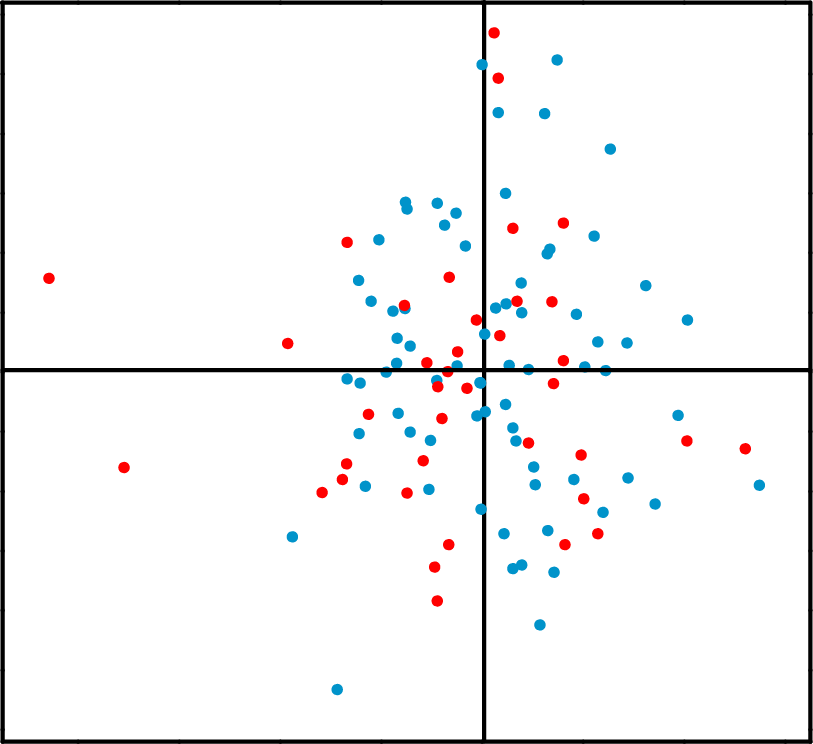


PC1 (3.87%)

PC2 (3.82%)

**Figure S1.** PCA from 20 microsatellite dataset of pygmy blue whales from the Australian feeding aggregations (red, Bonney Upwelling; blue, Perth Canyon).

**REFERENCES**

Attard CRM, Beheregaray LB, Jenner C, Gill P, Jenner M, Morrice M, Bannister J, LeDuc R, Möller L. 2010 Genetic diversity and structure of blue whales (*Balaenoptera musculus*) in Australian feeding aggregations. *Conserv. Genet.* **11**, 2437-2441. (doi:10.1007/s10592-010-0121-9)

Attard CRM, Beheregaray LB, Jenner KCS, Gill PC, Jenner M-N, Morrice MG, Robertson KM, Möller LM. 2012 Hybridization of Southern Hemisphere blue whale subspecies and a sympatric area off Antarctica: impacts of whaling or climate change? *Mol. Ecol.* **21**, 5715-5727. (doi:10.1111/mec.12025)

Attard CRM, Beheregaray LB, Jenner KCS, Gill PC, Jenner M-NM, Morrice MG, Teske PR, Möller LM. 2015 Low genetic diversity in pygmy blue whales is due to climate-induced diversification rather than anthropogenic impacts. *Biol. Lett.* **11**, 20141037. (doi:10.1098/rsbl.2014.1037)

Catchen J, Hohenlohe PA, Bassham S, Amores A, Cresko WA. 2013 Stacks: an analysis tool set for population genomics. *Mol. Ecol.* **22**, 3124-3140. (doi:10.1111/mec.12354)

Catchen JM, Amores A, Hohenlohe P, Cresko W, Postlethwait JH. 2011 Stacks: building and genotyping loci *de novo* from short-read sequences. *G3: Genes, Genomes, Genetics* **1**, 171-182. (doi:10.1534/g3.111.000240)

Coombs JA, Letcher BH, Nislow KH. 2008 CREATE: a software to create input files from diploid genotypic data for 52 genetic software programs. *Mol. Ecol. Resour.* **8**, 578-580. (doi:10.1111/j.1471-8286.2007.02036.x)

Earl DA, vonHoldt BM. 2012 STRUCTURE HARVESTER: a website and program for visualizing STRUCTURE output and implementing the Evanno method. *Conserv. Genet. Resour.* **4**, 359-361. (doi:10.1007/s12686-011-9548-7)

Evanno G, Regnaut S, Goudet J. 2005 Detecting the number of clusters of individuals using the software STRUCTURE: a simulation study. *Mol. Ecol.* **14**, 2611-2620. (doi:10.1111/j.1365-294X.2005.02553.x)

Excoffier L, Lischer HEL. 2010 Arlequin suite ver 3.5: a new series of programs to perform population genetics analyses under Linux and Windows. *Mol. Ecol. Resour.* **10**, 564-567. (doi:10.1111/j.1755-0998.2010.02847.x)

Falush D, Stephens M, Pritchard JK. 2003 Inference of population structure using multilocus genotype data: linked loci and correlated allele frequencies. *Genetics* **164**, 1567-1587.

Hubisz MJ, Falush D, Stephens M, Pritchard JK. 2009 Inferring weak population structure with the assistance of sample group information. *Mol. Ecol. Resour.* **9**, 1322-1332. (doi:10.1111/j.1755-0998.2009.02591.x)

Lischer HEL, Excoffier L. 2012 PGDSpider: an automated data conversion tool for connecting population genetics and genomics programs. *Bioinformatics* **28**, 298-299. (doi:10.1093/bioinformatics/btr642)

Peakall R, Smouse PE. 2006 GENALEX 6: genetic analysis in Excel. Population genetic software for teaching and research. *Mol. Ecol. Notes* **6**, 288-295. (doi:10.1111/j.1471-8286.2005.01155.x)

Peakall R, Smouse PE. 2012 GENALEX 6.5: genetic analysis in Excel. Population genetic software for teaching and research - an update. *Bioinformatics* **28**, 2537-2539. (doi:10.1093/bioinformatics/bts460)

Pritchard JK, Stephens M, Donnelly P. 2000 Inference of population structure using multilocus genotype data. *Genetics* **155**, 945-959.

Ryman N, Palm S. 2006 POWSIM: a computer program for assessing statistical power when testing for genetic differentiation. *Mol. Ecol.* **6**, 600-602. (doi:10.1111/j.1471-8286.2006.01378.x)
